# Novel modified blumgart anastomosis reduces clinically relevant pancreatic fistula after pancreaticoduodenectomy: a retrospective study using inverse probability of treatment weighting

**DOI:** 10.3389/fsurg.2025.1610561

**Published:** 2025-06-19

**Authors:** Lin Ye, Zhiyuan Jian, Wanrong Yue, Jun Weng, Qingrong Mo, Gaoshi Li, Renjian Li, Hao Shi, Haozhe Zhou, Yaqun Yu

**Affiliations:** ^1^ Department of Hepatobiliary and Pancreatic Surgery, Affiliated Hospital of Guilin Medical University, Guilin, China; ^2^ Department of Pathology, Guilin People's Hospital, Guilin, China; ^3^ College of Computer Science and Engineering, Guangxi Normal University, Guilin, China; ^4^ Department of Public Health, Guilin Medical University, Guilin, China

**Keywords:** pancreaticoduodenectomy, pancreaticojejunostomy, blumgart anastomosis, postoperative pancreatic fistula, inverse probability of treatment weighting

## Abstract

**Background:**

Clinically relevant postoperative pancreatic fistula (CR-POPF) remains a significant complication after pancreaticoduodenectomy (PD). We implemented a novel modified Blumgart pancreaticojejunostomy (m-BPJ) technique with anchoring approach and omental reinforcement, and evaluated its efficacy compared to conventional pancreaticojejunostomy (c-PJ).

**Methods:**

This retrospective study included patients who underwent PD from January 2020 to December 2024. Patients were divided into m-BPJ (*n* = 85) and c-PJ (*n* = 130) groups. Inverse probability of treatment weighting (IPTW) was applied to balance baseline characteristics. The primary endpoint was CR-POPF incidence. Secondary endpoints included operative parameters, postoperative recovery indicators, and complications.

**Results:**

After IPTW, CR-POPF incidence was significantly lower in the m-BPJ group (6.4% vs. 15.6%, *p* = 0.031). The m-BPJ group showed shorter PJ anastomosis time (21.1 ± 5.5 vs. 29.0 ± 7.4 min, *p* < 0.001), operation time (287.5 ± 45.3 vs. 304.2 ± 53.6 min, *p* = 0.023), and less intraoperative blood loss (325 vs. 375 mL, *p* = 0.041). Postoperative recovery was accelerated, with faster gastrointestinal function recovery (3.2 ± 1.1 vs. 4.0 ± 1.4 days, *p* < 0.001), earlier oral intake (4.6 ± 1.3 vs. 5.7 ± 1.8 days, *p* < 0.001), and reduced hospital stay (12 vs. 14 days, *p* = 0.009). Multivariate analysis confirmed m-BPJ as an independent protective factor against CR-POPF (OR 0.34, 95% CI 0.13-0.82, *p* = 0.018), while BMI ≥25 kg/m² (OR 2.23, 95% CI 1.07–4.65, *p* = 0.033), soft pancreatic texture (OR 3.25, 95% CI 1.47–7.12, *p* = 0.003), and pancreatic duct diameter <3 mm (OR 2.35, 95% CI 1.12–4.97, *p* = 0.024) were independent risk factors. Subgroup analysis revealed greatest benefit in high-risk patients.

**Conclusions:**

Our m-BPJ technique with anchoring approach and omental reinforcement significantly reduces CR-POPF after PD, particularly in high-risk patients. This technique demonstrates improved surgical efficiency and postoperative recovery, providing a valuable option for safer pancreatic reconstruction following PD.

## Introduction

Pancreaticoduodenectomy (PD) has remained the standard surgical procedure for treating benign and malignant lesions of the pancreatic head and periampullary region since it was first performed by Italian surgeon Codivilla in 1898 (1, 2). Gastrointestinal tract reconstruction following PD represents one of the most challenging aspects of the procedure and significantly influences patient recovery. While surgical mortality rates have decreased to below 5% over the past two decades (3–5). However, due to its complexity and intricate reconstruction steps, the postoperative complication rate remains high ranging from 30% to 60% (6–9).

Postoperative pancreatic fistula (POPF) represents a particularly concerning complication following PD. Despite not being the most frequent complication, clinically relevant POPF(CR-POPF) can significantly prolong hospitalization, increase healthcare costs, and potentially trigger life-threatening complications including post-pancreatectomy hemorrhage and intra-abdominal abscesses. Consequently, prevention and management of pancreatic fistula have become priorities in perioperative care (10).

In 2002, Blumgart from Memorial Sloan-Kettering Cancer Center developed an innovative pancreaticojejunostomy(PJ) technique for open PD that demonstrated promising clinical outcomes (11, 12). This approach has since been recognized as one of the safest anastomotic methods following PD (13, 14). inspiring various modifications across specialized pancreatic centers worldwide. However, consensus regarding the optimal modified Blumgart technique remains elusive.

In response to this challenge, we implemented a novel modified Blumgart PJ technique featuring an anchoring approach and omental reinforcement for open PD (15–18). This study aims to evaluate the efficacy of our modified technique compared to conventional PJ in reducing CR-POPF incidence. Secondary outcomes include operative duration, postoperative recovery, and mortality rates. To mitigate the inherent limitations of retrospective analysis, we employed inverse probability of treatment weighting (IPTW) to balance baseline characteristics between groups (19).

## Materials and methods

### Study design and patient selection

This retrospective study was conducted from January 2020 to December 2024 at the Department of Hepatobiliary and Pancreatic Surgery, Affiliated Hospital of Guilin Medical University. The study protocol was approved by the Ethics Committee of the Affiliated Hospital of Guilin Medical University (approval number: 2022YJSLL-95), and informed consent was obtained from all patients. The study was conducted in accordance with the Declaration of Helsinki and Good Clinical Practice guidelines.

**Figure 1:**
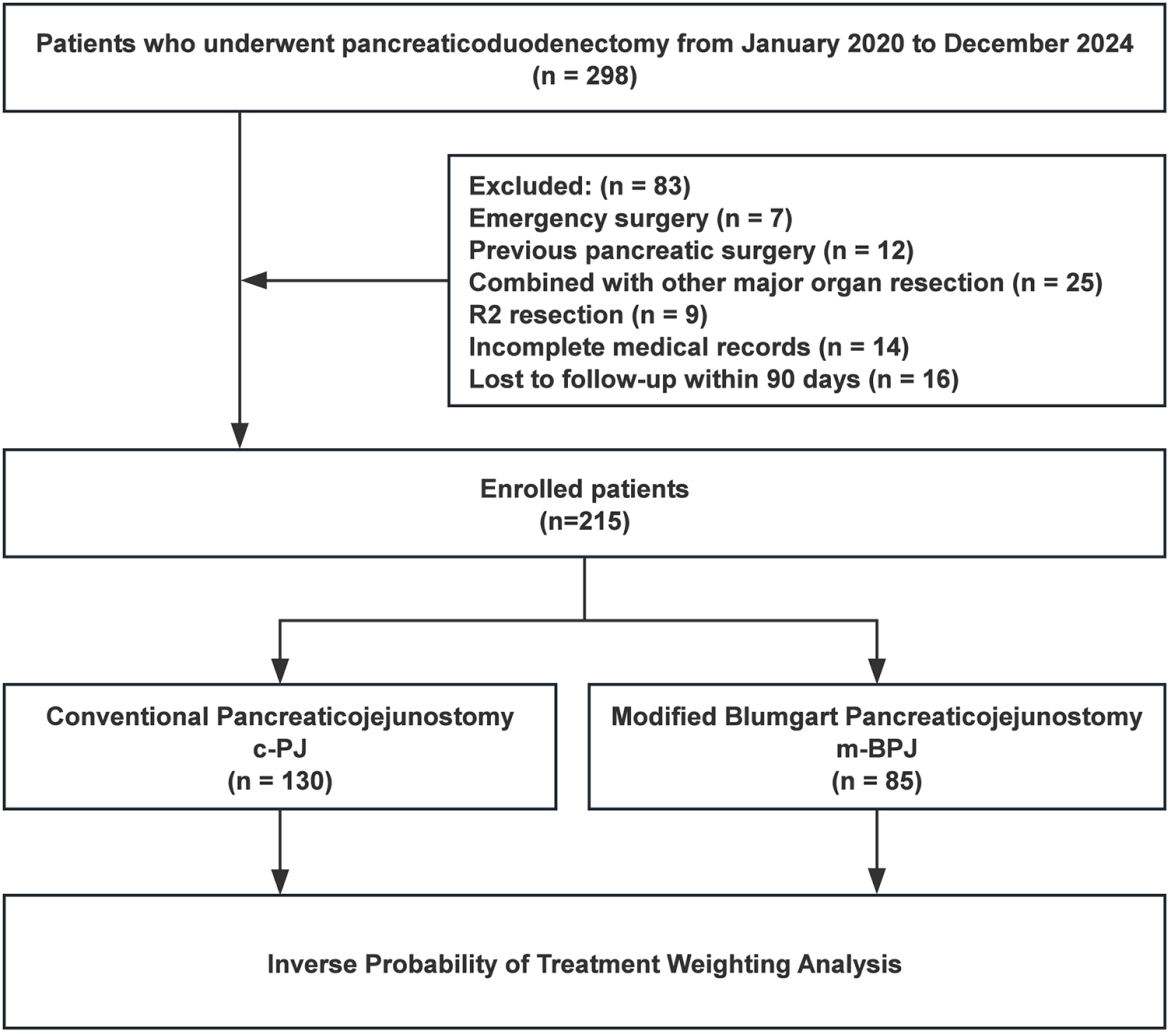
The flowchart of patient selection.

A total of 298 consecutive patients who underwent PD were initially screened for eligibility (Figure 1). Inclusion criteria were: (1) age ≥ 18 years; (2) elective PD for pathologically confirmed pancreatic head cancer, periampullary cancer, distal bile duct cancer, or duodenal cancer; (3) American Society of Anesthesiologists (ASA) physical status classification Ⅰ-ⅠⅡ; and (4) complete clinical, radiological, and follow-up data. Exclusion criteria were: (1) emergency surgery; (2) history of previous pancreatic surgery; (3) concurrent major organ resection; (4) macroscopically incomplete resection (R2); (5) incomplete medical records; (6) loss to follow-up within 90 days after surgery; (7) neoadjuvant therapy; (8) intraoperative finding of extensive metastasis precluding radical resection; (9) inability to tolerate general anesthesia; (10) severe coagulation disorders; and (11) pregnancy or lactation. (12) inability to place pancreatic duct stent due to technical factors.

**Figure 2:**
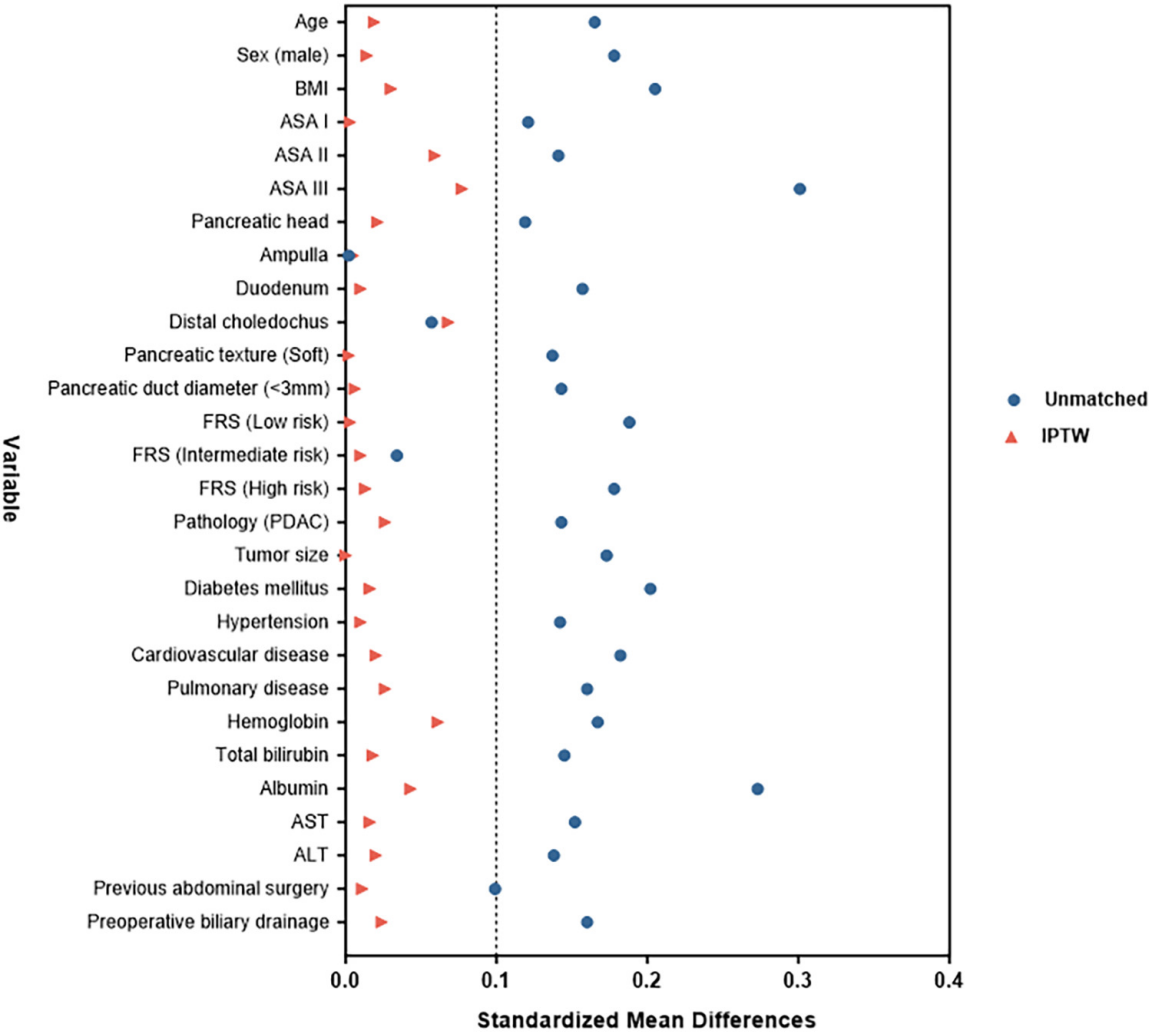
“Love” plot of standardized mean differences across the variables before and after IPTW. BMI, body mass index; ASA, American Society of Anesthesiologists; TBIL, Total Bilirubin; AST, aspartate aminotransferase; ALT, alanine aminotransferase.

After applying these criteria, patients were identified and divided into two groups based on the pancreaticojejunostomy anastomosis technique used: conventional PJ (c-PJ) group and modified Blumgart PJ (m-BPJ) group. To mitigate selection bias and balance the baseline characteristics between groups, inverse probability of treatment weighting (IPTW) based on propensity scores was applied. The propensity score was calculated using logistic regression with the following covariates: age, gender, body mass index, ASA classification, pancreatic texture (soft vs. hard), pancreatic duct diameter (<3 mm vs. ≥3 mm), Fistula Risk Score (FRS), pathology type (PDAC vs. non-PDAC), comorbidities (hypertension, diabetes mellitus, cardiovascular disease, pulmonary disease), previous abdominal surgery, lesion location, tumor size, preoperative biliary drainage, and preoperative laboratory values (hemoglobin, total bilirubin, albumin, aspartate aminotransferase, alanine aminotransferase) (Figure 2).

### Perioperative management

All patients received standardized perioperative management. Preoperative preparation included comprehensive assessment of nutritional status, with appropriate nutritional support provided for patients with albumin levels < 30 g/L. Routine preoperative evaluations included chest radiography, electrocardiography, and pulmonary function tests to assess surgical tolerance. Patients without iodine contrast allergy underwent thin-slice enhanced computed tomography of the upper abdomen to evaluate resectability and vascular variations. For patients with obstructive jaundice and total bilirubin levels exceeding 171 μmol/L, percutaneous transhepatic cholangial drainage or endoscopic retrograde cholangiopancreatography was routinely performed for biliary decompression.

Anesthesia consisted of general anesthesia combined with epidural anesthesia, with central venous pressure maintained at <5 cm H₂O during surgery to minimize bleeding. All patients received prophylactic intravenous antibiotics (second-generation cephalosporins) 30 min before incision, with an additional dose administered for operations exceeding 3 h. A modified fusiform incision was employed, and standard PD was performed. Hepaticojejunostomy and gastrojejunostomy were constructed via the retrocolic route, with single-layer interrupted sutures for hepaticojejunostomy and double-layer sutures for gastrojejunostomy.

Postoperatively, two intra-abdominal drains were placed in all patients: one adjacent to the PJ and the other near the hepaticojejunostomy. Drain fluid and serum amylase levels were measured on postoperative days 1, 3, 5, and 7. Liquid diet was initiated after flatus passage and in the absence of significant abdominal distension, nausea, or vomiting, with gradual progression to semi-liquid and regular diet. Postoperative analgesia was provided through epidural patient-controlled analgesia for 48–72 h. Nasogastric tube management followed a standardized protocol throughout the study period. All patients had nasogastric tubes placed intraoperatively for gastric decompression, which were routinely removed within 24–48 h postoperatively unless specific contraindications existed (such as delayed gastric emptying or excessive gastric residuals >200 ml per 8 h). Our center implemented a modified enhanced recovery after surgery (ERAS) protocol that included early nasogastric tube removal, early mobilization within 24 h, standardized multimodal analgesia, and early initiation of oral intake after return of bowel function. This standardized approach was applied consistently across both surgical groups to minimize confounding variables.

Criteria for drain removal included: drainage volume < 50 ml/day, drain fluid amylase < 3 times the normal serum value, and absence of active bleeding, bile leakage, or intestinal fistula. Drains were typically removed on postoperative days 5–7 in patients without complications. All patients participated in a standardized rehabilitation program, including early mobilization, respiratory exercises, and nutritional support. Discharge criteria comprised: afebrile status, satisfactory wound healing, tolerance of regular diet, no requirement for intravenous analgesia, and normalized laboratory parameters.

### Surgical technique

All procedures were performed by a single experienced hepatobiliary and pancreatic surgeon who had already completed over 200 pancreaticoduodenectomies prior to 2020 and the primary surgical assistants remained largely unchanged throughout the study period. The lead surgeon had reached the plateau of the learning curve for both techniques, minimizing any influence of technical proficiency on surgical outcomes. All operations were performed using an open approach with standardized protocols, including identical anesthesia methods, perioperative management, and postoperative care regimens.

Digestive tract reconstruction followed the Child's order, with PJ performed first, followed by hepaticojejunostomy and gastrojejunostomy. All anastomoses were established via the retrocolic route, with the jejunum brought behind the transverse colon for connection to the pancreas, bile duct, and stomach. During PJ, an appropriately sized internal stent was placed between the pancreatic duct and jejunal orifice without external drainage. This stent was designed to ensure smooth drainage of pancreatic juice and prevent early anastomotic obstruction.

In this study, we observed that the c-PJ was predominantly used from 2020 to 2022, while the m-BPJ was mainly implemented from 2023 to 2024. This transition in technique application reflects continuous optimization of surgical methods aimed at further reducing postoperative complications. While the fundamental steps and principles of both conventional and modified techniques were similar, they differed primarily in the anastomotic approach and reinforcement techniques. All patients received pancreatic duct stents and intra-abdominal drains, with postoperative monitoring of drain fluid amylase levels and clinical manifestations according to uniform standards.

### Conventional pancreaticojejunostomy (c-PJ)

From 2020 to 2022, we primarily employed the traditional end-to-side pancreaticojejunostomy technique described by Bassi (20, 21). In this technique, both the anterior and posterior walls were constructed using interrupted sutures, with 3–0 Vicryl sutures (Ethicon, Inc) fixing the pancreatic capsule and parenchyma to the seromuscular layer of the jejunum. Since the pancreatic duct typically occupies an eccentric dorsal position, its dorsal wall was also secured with 1–3 stitches passing through the pancreatic parenchyma to form the PJ. The anterior wall was subsequently sutured using the same interrupted suture technique, with intermediate stitches added when necessary. An appropriately sized internal stent was placed between the pancreatic duct and jejunal orifice without external drainage.

### Modified blumgart pancreaticojejunostomy (m-BPJ)

**Figure 3:**
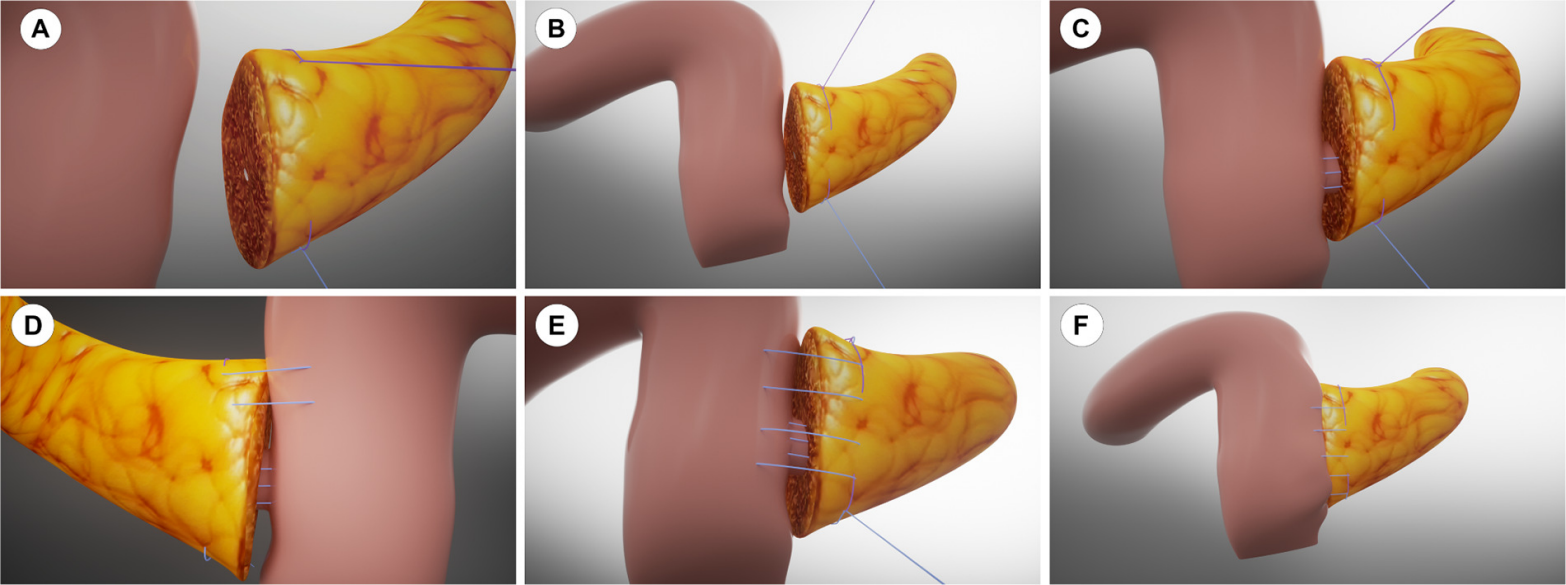
3D model of the modified Blumgart pancreaticojejunostomy with anchoring technique. **(A)** Pancreatic stump preparation. **(B)** Anterior “anchor line” establishment. **(C)** Pancreatic duct stent placement. **(D)** Horizontal mattress sutures placement lateral to the anchor line. **(E)** Completed suturing with knots tied. **(F)** Omental padding fixation.

**Figure 4:**
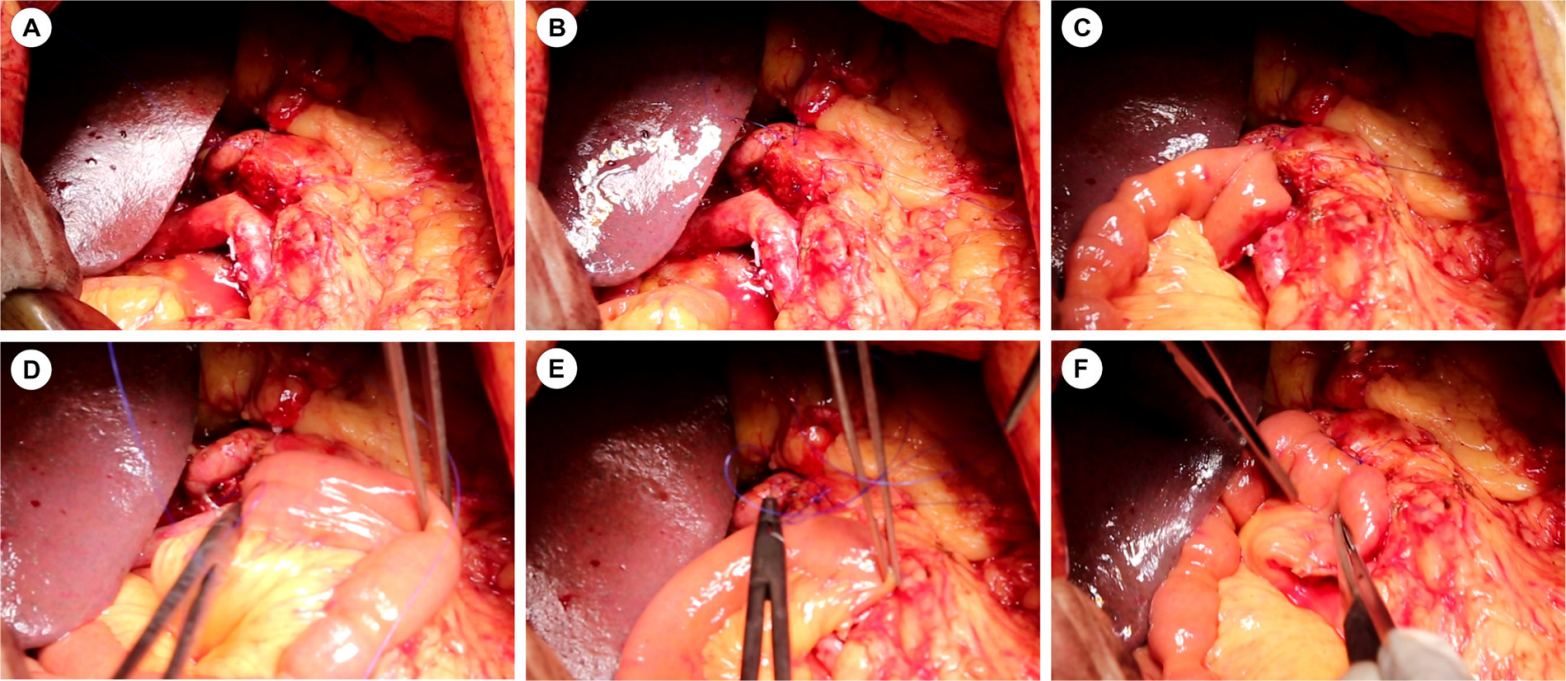
Intraoperative images of the modified Blumgart pancreaticojejunostomy technique. **(A–F)** Sequential surgical steps corresponding to the 3D model in Figures 3A–F.

From 2023 to 2024, we implemented our modified Blumgart PJ with omental reinforcement. As shown in Figure 3 and Figure 4 (3D models and animation created with Cinema 4D R25, Maxon Computer GmbH, Friedrichsdorf, Germany, available in Supplementary Materials). The procedure begins with transecting the pancreatic neck and mobilizing the pancreatic remnant 1–2 cm towards the tail. The pancreatic stump is then sutured using 3–0 Vicryl(Ethicon, Inc), leaving the sutures untied for traction and fixation (Figures 3A, 4A). An anterior “anchor line” is established along the pancreatic cut surface's serosal edge, creating differential tension between the dorsal and ventral sides of the anastomosis (Figures 3B, 4B). The main pancreatic duct is identified, and an appropriately sized stent is inserted and secured with continuous 5–0 PDS Ⅱ(Ethicon, Inc) sutures. The stent's other end is passed through a small hole in the jejunal seromuscular layer and sutured to the serosa, completing the duct anastomosis (Figures 3C, 4C). The PJ is then reinforced using two-layer horizontal mattress sutures, which are placed from one side of the pancreatic stump, through the entire thickness of the pancreatic tissue along the anterior “anchor line”, and into the jejunal seromuscular layer (Figures 3D, 4D).This process is repeated 3–4 times depending on the stump size, with the sutures finally tied off from the opposite side of the pancreatic stump (Figures 3E, 4E). A unique feature of our technique is the addition of a suitable-sized vascularized piece of the greater omentum, which is cut and passed behind the PJ, fixed with Hem-o-lok clips or silk sutures to prevent contact between the anastomosis and the posteriorly exposed vessels (celiac trunk, common hepatic artery, superior mesenteric artery, and superior mesenteric vein) (Figures 3F, 4F).

In both techniques, all patients received pancreatic duct stents and intra-abdominal drains, with postoperative monitoring of drain fluid amylase levels and clinical manifestations according to uniform standards.

### Study endpoints and definitions

The primary endpoint of this study was the incidence of clinically relevant postoperative pancreatic fistula (CR-POPF), defined as grade B or C pancreatic fistula according to the 2016 update of the International Study Group on Pancreatic Fistula (ISGPF)classification (22). Grade B POPF requires a change in postoperative management (such as drain retention beyond 3 weeks or repositioning through percutaneous or endoscopic procedures), while Grade C POPF necessitates reoperation or results in single/multiple organ failure or death. Secondary endpoints encompassed operative parameters (total operation time, PJ anastomosis time, and intraoperative blood loss), postoperative recovery indicators (time to gastrointestinal function recovery, time to oral intake, and length of hospital stay), and complications occurring within 90 days after surgery. All complications were classified according to the Clavien-Dindo system, with grade ≥ III defined as severe complications (23). The assessed complications primarily included biochemical leak (amylase level in drain fluid ≥ 3 times the upper normal serum value on or after postoperative day 3, without clinical significance), postoperative hemorrhage (as defined by the International Study Group of Pancreatic Surgery), bile leakage (bilirubin concentration in drain fluid day ≥ 3 times the serum bilirubin concentration on or after postoperative day 3), delayed gastric emptying (according to ISGPS criteria), pulmonary infection, intra-abdominal infection, and reoperation. Additionally, 30-day readmission rate and 90-day mortality rate were recorded. For risk factor analysis, predetermined cutoff values were used to evaluate variables including surgical technique, age, gender, BMI, diabetes history, jaundice severity, preoperative albumin level, pancreatic texture, pancreatic duct diameter, and intraoperative blood loss for their impact on CR-POPF occurrence.

### Statistical analysis

Statistical analyses were performed using SPSS version 26.0 (IBM Corp., Armonk, NY, USA) and R version 4.1.0 (R Foundation for Statistical Computing, Vienna, Austria). Continuous variables were presented as the mean and standard deviation or median (interquartile range) and compared using the Student's *t*-test or Mann–Whitney *U*-test, as appropriate. Categorical variables were presented as frequencies (percentages) and compared using the chi-square test or Fisher's exact test.

To minimize potential confounders, inverse probability of treatment weighting (IPTW) was performed between the treatment groups (24). After weighting, balance between covariates was assessed using standardized mean differences, with a threshold of <0.1 indicating good balance. Univariate and multivariate logistic regression analyses were conducted to identify risk factors for CR-POPF. Variables with *p* < 0.1 in univariate analysis were included in the multivariate model. For the analysis of multiple secondary endpoints, we applied Bonferroni correction to control the family-wise error rate, adjusting the significance threshold to *p* < 0.005 (0.05/10) for these outcomes. For other analyses, a two-sided *p* value < 0.05 was considered statistically significant.

## Results

### Clinical characteristics and preoperative Status

Table 1 presents the demographic characteristics, clinical features, and laboratory findings of the m-BPJ and c-PJ groups. Before IPTW, the two groups showed no significant differences in age (57.3 ± 11.2 vs. 59.1 ± 10.5 years, *p* = 0.225), gender distribution (male: 57.6% vs. 66.2%, *p* = 0.174), and BMI (23.1 ± 3.2 vs. 23.8 ± 3.5 kg/m², *p* = 0.133). However, ASA classification demonstrated a statistically significant difference between groups (*p* = 0.042), with the modified group having higher proportions of class Ⅰ and Ⅱ patients (classⅠ: 16.5% vs. 12.3%; class Ⅱ: 74.1% vs. 67.7%) and a lower proportion of class III patients (9.4% vs. 20.0%), suggesting potentially better overall condition in the modified group.

**Table 1:** Demographic, clinical characteristics and laboratory findings.

Characteristic	Before IPTW			After IPTW		
	c-PJ (*n* = 130)	m-BPJ (*n* = 85)	*p* value	c-PJ (*n* = 122)	m-BPJ (*n* = 94)	*p* value
Age (years), mean ± SD	59.1 ± 10.5	57.3 ± 11.2	0.225	58.3 ± 10.7	58.1 ± 10.8	0.893
Sex, *n* (%)			0.174			0.852
Male	86 (66.2)	49 (57.6)		77 (63.4)	60 (64.2)	
Female	44 (33.8)	36 (42.4)		45 (36.9)	34 (36.2)	
BMI (kg/m2), mean ± SD	23.8 ± 3.5	23.1 ± 3.2	0.133	23.4 ± 3.4	23.5 ± 3.3	0.836
ASA classification, *n* (%)			**0**.**042**			0.764
Ⅰ	16 (12.3)	14 (16.5)		18 (14.8)	13 (13.9)	
Ⅱ	88 (67.7)	63 (74.1)		85 (70.0)	70 (71.6)	
Ⅲ	26 (20.0)	8 (9.4)		19 (15.6)	13 (13.8)	
Lesion location, *n* (%)			0.483			0.914
Pancreatic head	87 (66.9)	52 (61.2)		78 (64.2)	61 (65.2)	
Ampulla	29 (22.3)	19 (22.4)		27 (22.2)	21 (22.5)	
Duodenum	8 (6.2)	9 (10.6)		10 (8.2)	7 (7.5)	
Distal choledochus	6 (4.6)	5 (5.9)		7 (5.7)	5 (5.3)	
Pancreatic texture, *n* (%)			0.245			0.903
Soft	85 (65.4)	61 (71.8)		83 (68.0)	64 (68.1)	
Hard	45 (34.6)	24 (28.2)		39 (32.0)	30 (31.9)	
Pancreatic duct diameter, *n* (%)			0.183			0.968
<3 mm	72 (55.4)	53 (62.4)		71 (58.2)	55 (58.5)	
≥3 mm	58 (44.6)	32 (37.6)		51 (41.8)	39 (41.5)	
Fistula Risk Score, *n* (%)			0.196			0.943
Low risk (0–2)	24 (18.5)	10 (11.8)		18 (14.8)	14 (14.9)	
Intermediate risk **(**3–6)	68 (52.3)	43 (50.6)		63 (51.6)	49 (52.1)	
High risk (7–10)	38 (29.2)	32 (37.6)		41 (33.6)	31 (33.0)	
Tumor size (cm), mean ± SD	3.4 ± 1.2	3.2 ± 1.1	0.201	3.3 ± 1.2	3.3 ± 1.1	0.985
Comorbidity, *n* (%)						
Diabetes mellitus	41 (31.5)	19 (22.4)	0.140	35 (28.7)	22 (23.4)	0.865
Hypertension	58 (44.6)	32 (37.6)	0.307	53 (43.4)	34 (36.2)	0.912
Cardiovascular disease	22 (16.9)	9 (10.6)	0.194	17 (13.9)	10 (10.6)	0.773
Pulmonary disease	17 (13.1)	7 (8.2)	0.271	13 (10.7)	8 (8.5)	0.881
Laboratory findings						
Hemoglobin (g/L), median (IQR)	125 (114–138)	128 (118–140)	0.232	126 (115–139)	127 (116–139)	0.621
TBIL (umol/L), median (IQR)	48.5 (18.3–127.8)	36.4 (15.2–102.5)	0.295	42.7 (17.5–114.6)	43.5 (16.9–110.3)	0.897
Albumin (g/L), median (IQR)	36.2 (32.5–40.8)	37.5 (34.2–41.6)	0.056	36.8 (33.5–41.0)	37.0 (33.6–40.8)	0.761
AST (U/L), median (IQR)	42 (28–78)	39 (24–65)	0.216	41 (26–74)	40 (25–68)	0.879
ALT (U/L), median (IQR)	57 (32–96)	53 (28–88)	0.328	55 (30–92)	54 (29–90)	0.851
Previous abdominal surgery, *n* (%)	23 (17.7)	12 (14.1)	0.482	19 (15.6)	15 (16.0)	0.932
Preoperative biliary drainage, *n* (%)	43 (33.1)	22 (25.9)	0.256	35 (28.7)	28 (29.8)	0.851

c-PJ, conventional pancreaticojejunostomy anastomosis; m-BPJ, modified Blumgart pancreaticojejunostomy anastomosis; IPTW, inverse probability of treatment weighting; SD, standard deviation; BMI, body mass index; ASA, American society of anesthesiologists; TBIL, total bilirubin; AST, aspartate aminotransferase; ALT, alanine aminotransferase; IQR, interquartile range. Bold indicates statistical significance (*p* < 0.05).

Regarding comorbidities, there were no significant differences in hypertension, diabetes, coronary heart disease, and chronic pulmonary disease between groups. The rates of previous abdominal surgery were also comparable (14.1% vs. 17.7%, *p* = 0.482). Tumor location distribution was similar between groups (*p* = 0.483), with pancreatic head cancer being the predominant type (61.2% vs. 66.9%), followed by periampullary cancer, distal bile duct cancer and duodenal cancer. Tumor size (*p* = 0.201) and preoperative biliary drainage rates (*p* = 0.256) also showed no significant differences between groups.

For pancreatic risk factors, pancreatic texture (soft: 71.8% vs. 65.4%, *p* = 0.245) and pancreatic duct diameter (<3 mm: 62.4% vs. 55.4%, *p* = 0.183) showed no significant differences between groups. The distribution of FRS was also similar (*p* = 0.196), although the m-BPJ group had a slightly higher proportion of high-risk patients (FRS 7–10: 37.6% vs. 29.2%). Pathology types were comparable between groups, with non-PDAC pathology being more prevalent in both groups (62.4% vs. 55.4%, *p* = 0.276).

In laboratory tests, albumin levels in the modified group approached statistical significance compared to the conventional group (*p* = 0.056), while hemoglobin, total bilirubin AST and ALT showed no significant differences between groups.

After applying IPTW, all baseline characteristics achieved good balance between groups, with allp-values > 0.7 and all standardized mean differences < 0.1, indicating successful elimination of potential selection bias and providing a reliable foundation for subsequent outcome analysis.

### Perioperative details and postoperative complications

Table 2 presents a comparison of postoperative outcomes between the m-BPJ and c-PJ groups. Before IPTW, only time to gastrointestinal function recovery (3.3 ± 1.2 vs. 4.0 ± 1.3 days, *p* = 0.003), time to start oral intake (4.7 ± 1.4 vs. 5.6 ± 1.7 days, *p* = 0.005), and PJ anastomosis time (21.5 ± 5.8 vs. 28.7 ± 7.3 min, *p* < 0.001) showed significant differences between the groups.

**Table 2:** Perioperative findings and postoperative outcomes.

Characteristic	Before IPTW			After IPTW		
	c-PJ (*n* = 130)	m-BPJ (*n* = 85)	*p* value	c-PJ (*n* = 122)	m-BPJ (*n* = 94)	*p* value
Operative indicators						
Operative time (min), mean ± SD	302.6 ± 51.9	290.3 ± 47.8	0.082	303.8 ± 53.1	289.2 ± 45.5	**0**.**023**
PJ-time (min), mean ± SD	28.7 ± 7.3	21.5 ± 5.8	<**0**.001**	29.0 ± 7.4	21.1 ± 5.5	**<0**.001**
Blood loss (ml), median (IQR)	370 (250- 510)	350 (220- 480)	0.118	375 (255–515)	325 (215–455)	**0**.**041**
Postoperative recovery						
Time to gastrointestinal function recovery(d), mean ± SD	4.0 ± 1.3	3.3 ± 1.2	**0**.003**	4.0 ± 1.4	3.2 ± 1.1	<**0**.001**
Time to start oral intake(d), mean ± SD	5.6 ± 1.7	4.7 ± 1.4	**0**.005**	5.7 ± 1.8	4.6 ± 1.3	<**0**.001**
Length of hospital stay(d), median(IQR)	14 (11–17)	13 (10–16)	0.065	14 (12–18)	12 (10–15)	**0**.**009**
Postoperative complications, *n* (%)						
Clavien-Dindo grade ≥ Ⅲ	19 (4.6)	10 (11.8)	0.552	20 (16.4)	9 (9.6)	**0**.**046**
Pancreatic fistula						
Biochemical leak	36 (27.7)	22 (25.9)	0.764	37 (30.3)	22 (23.4)	0.251
Grade B	15 (11.5)	7 (8.2)	0.430	16 (13.1)	5 (5.3)	**0**.**042**
Grade C	2 (1.5)	1 (1.2)	0.825	3 (2.5)	1 (1.1)	0.541
CR-POPF	17 (13.1)	8 (9.4)	0.408	19 (15.6)	6 (6.4)	**0**.**031**
Hemorrhage	7 (5.4)	4 (4.7)	0.827	8 (6.6)	3 (3.2)	0.388
Bile leakage	5 (3.8)	2 (2.4)	0.705	5 (4.1)	2 (2.1)	0.411
Delayed gastric emptying	17 (13.1)	8 (9.4)	0.408	18 (14.8)	7 (7.4)	**0**.**039**
Pulmonary infection	12 (9.2)	6 (7.1)	0.567	13 (10.7)	5 (5.3)	0.151
Intra-abdominal infection	10 (7.7)	5 (5.9)	0.602	11 (9.0)	4 (4.3)	0.173
Reoperation	8 (6.2)	3 (3.5)	0.536	9 (7.4)	3 (3.2)	0.169
30 day readmission rate, *n* (%)	12 (9.2)	6 (7.1)	0.567	13 (10.7)	5 (5.3)	0.151
90 days mortality rate, *n* (%)*	3 (2.3)	1 (1.2)	0.649	3 (2.5)	1 (1.1)	0.432

* 90-day mortality was defined as death from any cause within 90 days after surgery. Bold indicates statistical significance (*p* < 0.05).

** Remains significant after Bonferroni correction for multiple testing (*p* < 0.005).

PJ, pancreaticojejunostomy; POPF, postoperative pancreatic fistula; CR-POPF, clinically relevant postoperative pancreatic fistula; IPTW, inverse probability of treatment weighting; SD, standard deviation; IQR, interquartile range.

After balancing baseline characteristics with IPTW, significant differences emerged in several additional outcomes. The m-BPJ group demonstrated shorter operation time (287.5 ± 45.3 vs. 304.2 ± 53.6 min, *p* = 0.023). Although this reduction was modest in absolute terms (16.7 min or 5.5%), it primarily reflects the significantly shorter PJ anastomosis time, which is particularly relevant for reducing warm ischemia time of the pancreatic remnant. The advantage in PJ anastomosis time remained highly significant (21.1 ± 5.5 vs. 29.0 ± 7.4 min, *p* < 0.001), reflecting a 27.2% reduction in the time required to complete the anastomosis. The m-BPJ group also showed less intraoperative blood loss [325 [215–455] vs. 375 [255–515] ml, *p* = 0.041].

The m-BPJ group maintained significant advantages in time to gastrointestinal function recovery (3.2 ± 1.1 vs. 4.0 ± 1.4 days, *p* < 0.001) and time to start oral intake (4.6 ± 1.3 vs. 5.7 ± 1.8 days, *p* < 0.001). Additionally, after IPTW, a significant difference in length of hospital stay became apparent [12 [10–15] vs. 14 [12–18] days, *p* = 0.009]. The incidence of severe postoperative complications (Clavien-Dindo grade ≥ III) was significantly lower in the m-BPJ group after IPTW (9.6% vs. 16.4%, *p* = 0.046). Notably, the rate of clinically relevant (grade B/C) POPF was significantly reduced in the m-BPJ group (6.4% vs. 15.6%, *p* = 0.031), representing a 59.0% relative reduction. This improvement was primarily driven by a significant reduction in Grade B pancreatic fistula (5.3% vs. 13.1%, *p* = 0.042), while Grade C fistula rates remained low in both groups (1.1% vs. 2.5%, *p* = 0.432). Similarly, delayed gastric emptying occurred less frequently in the m-BPJ group (7.4% vs. 14.8%, *p* = 0.039).

After applying Bonferroni correction (adjusted *p*-value threshold <0.005) to control for multiple testing, PJ anastomosis time (*p* < 0.001), time to gastrointestinal function recovery (*p* < 0.001), and time to start oral intake (*p* < 0.001) remained significantly different between groups, while other secondary outcomes including operation time, blood loss, length of hospital stay, and postoperative complications did not meet the stricter significance threshold, suggesting these results should be interpreted with caution.

Other complications, including biochemical leak, hemorrhage, bile leakage, pulmonary infection, intra-abdominal infection, and reoperation, showed lower incidence in the m-BPJ group, but the differences did not reach statistical significance after IPTW. The 30-day readmission rate (5.9% vs. 10.0%, *p* = 0.115) and 90-day mortality (1.2% vs. 2.3%, *p* = 0.541) were also comparable between the two groups.

### Predictive factors for clinically relevant POPF

To identify risk factors for CR-POPF, we performed univariate and multivariate logistic regression analyses on the IPTW-weighted data (Table 3). Univariate analysis revealed that the modified technique was significantly associated with reduced risk of CR-POPF (OR: 0.37, 95% CI: 0.15–0.89, *p* = 0.031). Concurrently, BMI ≥ 25 kg/m² (OR: 2.42, 95% CI: 1.18–4.95, *p* = 0.016), soft pancreatic texture (OR: 3.58, 95% CI: 1.68–8.15, *p* = 0.001), and pancreatic duct diameter < 3 mm (OR: 2.63, 95% CI: 1.26–5.57, *p* = 0.009) were identified as significant risk factors for CR-POPF, consistent with our subgroup analysis findings. Non-PDAC pathology also showed a trend toward increased risk (OR: 1.88, 95% CI: 0.98–3.76, *p* = 0.063). Additionally, preoperative albumin < 35 g/L (OR: 1.91, 95% CI: 0.92–3.98, *p* = 0.082) and intraoperative blood loss ≥ 500 ml (OR: 1.92, 95% CI: 0.93–3.95, *p* = 0.076) demonstrated marginal significance. However, age ≥ 65 years, gender, diabetes, and jaundice showed no significant association with CR-POPF occurrence.

**Table 3:** Risk factors for clinically relevant postoperative pancreatic fistula after IPTW.

Variable	Univariable			Multivariable		
	OR	95% CI	*p* value	OR	95% CI	*p* value
Surgical technique						
Conventional	Reference	Reference		Reference	Reference	
Modified	0.37	(0.15–0.89)	**0**.**031**	0.34	0.13–0.82	**0**.**018**
Age (≥65 years)	1.35	0.62–2.98	0.452			
Sex (male)	0.85	0.38–1.87	0.683			
BMI (≥25 kg/m^2^)	2.42	1.18–4.95	**0**.**016**	2.23	1.07–4.65	**0**.**033**
Diabetes mellitus	1.19	0.51–2.75	0.687			
Jaundice (total bilirubin ≥ 34.2 μmol/L)	1.52	0.78–3.15	0.263			
Preoperative albumin < 35 g/L	1.91	0.92–3.98	0.082	1.65	0.76–3.52	0.195
Pancreatic texture						
Hard	Reference	Reference		Reference	Reference	
Soft	3.58	1.68–8.15	**0**.**001**	3.25	1.47–7.12	**0**.**003**
Pancreatic duct diameter < 3 mm	2.63	1.26–5.57	**0**.**009**	2.35	1.12–4.97	**0**.**024**
Intraoperative blood loss ≥ 500 ml	1.92	0.93–3.95	0.076	1.62	0.78–3.42	**0**.**198**

OR, odds ratio; CI, confidence interval. Bold indicates statistical significance (*p* < 0.05).

When variables with *p* < 0.1 were included in the multivariate regression model, the modified technique was confirmed as an independent protective factor against CR-POPF (OR: 0.34, 95% CI: 0.13–0.82, *p* = 0.018), suggesting this technique could reduce CR-POPF risk by 66%. Meanwhile, BMI ≥25 kg/m² (OR: 2.23, 95% CI: 1.07–4.65, *p* = 0.033), soft pancreatic texture (OR: 3.25, 95% CI: 1.47–7.12, *p* = 0.003), and pancreatic duct diameter < 3 mm (OR: 2.35, 95% CI: 1.12–4.97, *p* = 0.024) were verified as independent risk factors for CR-POPF. Non-PDAC pathology approached statistical significance (OR: 1.79, 95% CI: 0.89–3.62, *p* = 0.102). In the multivariate analysis, preoperative albumin level and intraoperative blood loss did not demonstrate statistical significance.

These findings indicate that, after controlling for known risk factors, the modified technique exhibits a clear and independent protective effect in reducing CR-POPF risk. The significant associations between CR-POPF and established risk factors such as soft pancreatic texture, small pancreatic duct diameter, and higher BMI in our analysis are consistent with previous literature. The fact that the modified technique remained protective even after adjusting for these known risk factors suggests that the technical modifications themselves, rather than patient selection or other confounding factors, are responsible for the observed reduction in CR-POPF. This provides support for the efficacy of our modified approach in PD.

### Subgroup analysis

Subgroup analyses based on pancreatic characteristics (Table 4) revealed that the protective effect of the modified technique was particularly pronounced in high-risk patients with soft pancreatic texture (CR-POPF: 9.4% vs. 21.7%, *p* = 0.011) and small pancreatic duct diameter <3 mm (CR-POPF: 10.9% vs. 22.5%, *p* = 0.042). Patients with high FRS scores (7–10) showed the most significant benefit from the modified technique (CR-POPF: 9.7% vs. 26.8%, *p* = 0.038).

**Table 4:** Subgroup analysis of CR-POPF rates based on pancreatic characteristics after IPTW.

Subgroup	c-PJ	m-BPJ	*p* value
Pancreatic texture			
Soft	18/83 (21.7%)	6/64 (9.4%)	**0** **.** **011**
Hard	1/39 (2.6%)	0/30 (0.0%)	0.376^†^
Pancreatic duct diameter			
<3 mm	16/71 (22.5%)	6/55 (10.9%)	**0**.**042**
≥3 mm	3/51 (5.9%)	0/39 (0.0%)	0.126^†^
Fistula Risk Score			
Low risk (0–2)	1/18 (5.6%)	0/14 (0.0%)	0.370^†^
Intermediate risk (3–6)	7/63 (11.1%)	3/49 (6.1%)	0.245
High risk (7–10)	11/41 (26.8%)	3/31 (9.7%)	**0**.**038**
Pathology			
PDAC	5/49 (10.2%)	2/39 (5.1%)	0.284
Non-PDAC	14/73 (19.2%)	4/55 (7.3%)	**0**.**045**
Intraoperative blood loss			
<400 ml	11/82 (13.4%)	3/67 (4.5%)	**0**.**047**
≥400 ml	8/40 (20.0%)	3/27 (11.1%)	0.265
BMI			
<25 kg/m²	10/78 (12.8%)	3/63 (4.8%)	0.082
≥25 kg/m²	9/44 (20.5%)	3/31 (9.7%)	0.142

^†^ Fisher's exact test was used for groups with expected cell counts less than 5. Bold indicates statistical significance (*p* < 0.05).

c-PJ, conventional pancreaticojejunostomy; m-BPJ, modified Blumgart pancreaticojejunostomy; CR-POPF, clinically relevant postoperative pancreatic fistula; IPTW, inverse probability of treatment weighting.

Additionally, non-PDAC pathology patients (CR-POPF: 7.3% vs. 19.2%, *p* = 0.045) and those with intraoperative blood loss <400 ml (CR-POPF: 4.5% vs. 13.4%, *p* = 0.047) demonstrated significant reduction in CR-POPF rates with the modified technique. In contrast, patients with hard pancreatic texture or larger duct diameter showed low CR-POPF rates regardless of the anastomotic technique used, suggesting that the modified technique provides the greatest benefit specifically for high-risk patients.

## Discussion

This study compares the clinical outcomes of our center's Modified Blumgart Anastomosis technique vs. conventional technique in PD. After balancing baseline characteristics between groups through IPTW, we found that the modified technique significantly reduced the incidence of clinically relevant pancreatic fistula (CR-POPF) while improving multiple perioperative indicators. The CR-POPF rate was 6.4% in the modified group compared to 15.6% in the conventional group (*p* = 0.031), and multivariate analysis confirmed that the modified technique was an independent protective factor against CR-POPF (OR: 0.34, 95% CI: 0.13–0.82, *p* = 0.018). Subgroup analysis further revealed that this protective effect was particularly pronounced in high-risk patients with soft pancreatic texture and small pancreatic duct diameter.

PD is the gold standard surgical procedure for treating malignant tumors of the pancreatic head, periampullary region, distal bile duct, and duodenum. However, despite continuous advancements in surgical techniques, POPF remains one of the most challenging complications of this procedure. POPF is the most significant postoperative complication following PD, with incidence rates varying widely across different centers, ranging from 11.4% to 41.7% (25–27). This complication is particularly dangerous as it can lead to intra-abdominal infection and hemorrhage, is closely associated with poor prognosis, and carries a mortality rate of up to 9% (28). In clinical practice, CR-POPF has greater clinical significance, with literature reports indicating that its incidence consistently remains at a high level of 10%–15% (29–31). In our study, the CR-POPF incidence rate in the traditional surgery group was 15.6%, consistent with published literature. However, with the application of our modified Blumgart PJ technique, the incidence rate was significantly reduced to 6.4%, representing a 59.0% reduction. This improvement provides new technical support for enhancing the safety of PD and has important clinical application value.

Since Blumgart first introduced his method, surgeons worldwide have continuously refined and optimized this technique to address various clinical needs. The results of this study demonstrate that modified techniques effectively reduce the incidence of CR-POPF, which aligns with the overall trend in the development of PJ techniques. In a 2023 study by Liu et al., a novel modified Blumgart anastomosis technique was applied to laparoscopic PD with promising results, achieving a CR-POPF rate of just 9.1% and an average PJ anastomosis time of approximately 30 min (32).Their modified approach featured strategic simplifications including purse-string sutures to create a fixed sinus between the pancreatic duct and jejunum mucosa, along with intermittent interlocking U-shaped sutures designed to closely approximate the jejunal serosa to the pancreatic stump while minimizing dead space formation. Similarly, in Kalev's study, their modified Blumgart technique utilized two transpancreatic mattress sutures with double-armed monofilament PDS 3–0, followed by PJ with 4 PDS 5–0 sutures, have the potential to decrease major POPF (21).

However, some studies suggest that the anastomotic technique may not be the primary determinant of POPF development. As demonstrated in the single-center propensity score matching analysis by Bellotti et al. (33), rates of CR-POPF did not differ significantly between three different reconstruction techniques: Neuhaus-style telescope pancreatojejunostomy (16%), pancreatogastrostomy (17%), and modified Blumgart-style PJ (15%), with no significant differences in matched analysis (*p* = 0.901). Their findings suggest “no crucial role of the applied reconstruction technique” in CR-POPF development. This conclusion is further supported by Hirono et al.'s randomized controlled trial, which found that the modified Blumgart mattress suture technique did not reduce CR-POPF compared with interrupted suture (10.3% vs. 6.8%; *p* = 0.367), despite achieving better technical outcomes such as shorter anastomosis time and smaller interspace between the pancreatic cut surface and jejunal wall (34). The controversy regarding optimal PJ technique remains active in current surgical literature. Multiple randomized controlled trials are underway to provide higher-level evidence on this topic. Nevertheless, our center's experience, as demonstrated in the present study, indicates that our modified approach has yielded tangible benefits for our patient population. The significant reduction in CR-POPF rates observed in our cohort suggests that, while patient and disease factors certainly play important roles, technical refinements can still contribute to improved outcomes in PD procedures. This is particularly relevant in our specific patient demographic and clinical setting, where the modified technique appears to address the particular challenges we commonly encounter.

The mechanism by which our center's modified technique reduces CR-POPF incidence likely involves multiple aspects. The anchoring suture technique represents a critical innovation, particularly beneficial for soft pancreatic tissue. By establishing anchoring points on the anterior wall of the pancreatic stump using 3–0 Vicryl, this approach significantly reduces tension on the anterior pancreatic wall-crucial for preventing fistula formation. In cases with soft pancreatic texture, traditional suturing methods often lead to tissue tearing, whereas anchoring sutures protect tissue integrity by preventing excessive longitudinal cutting of the pancreatic parenchyma, thereby reducing the likelihood of needle-hole leakage. This modified technique achieves secure anastomosis even with fragile pancreatic tissue, improving surgical success rates and reducing postoperative complications. Furthermore, continuous suturing of the pancreatic stump to the jejunum offers distinct advantages over the interrupted sutures used in traditional Blumgart PJ. Employing continuous mattress suturing lateral to the anchoring points not only significantly reduces operative time but, when combined with anchoring sutures, minimizes the shearing force during knot-tying and reduces the risk of suture-induced pancreatic parenchymal damage. In cases with soft pancreatic texture, traditional interrupted suturing may cause uneven force distribution around each suture point, whereas continuous mattress suturing evenly distributes pressure, reducing the risk of pancreatic parenchymal tearing. Additionally, this technique reduces the number of sutures required, simplifies the procedure, and enhances overall anastomotic efficiency. The significant reduction in PJ time (21.1 ± 5.5 vs. 29.0 ± 7.4 min, *p* < 0.001) decreases tissue ischemia and edema, potentially promoting earlier anastomotic healing.

As an important component of the modified Blumgart PJ, this technique innovatively applies omental padding by cutting an appropriately sized piece of vascularized greater omentum, passing it through the posterior aspect of the PJ anastomosis, and securing it to the remnant hepatoduodenal ligament using Hem-o-lok clips or sutures. The greater omentum, as a critical abdominal barrier, possesses multiple physiological functions including promoting anti-infection processes, enhancing immune response, and secreting and absorbing peritoneal fluid (35–37). Additionally, the omentum effectively regulates gastrointestinal circulation, delivers vascular endothelial growth factors, accelerates new vessel formation at the pancreaticojejunal anastomosis site, and improves local blood supply (38).

While our hypothesis that omental padding reduces CR-POPF by isolating the anastomosis from pancreatic juice is mechanistically plausible, we acknowledge the lack of direct imaging or reoperation evidence supporting this specific mechanism in our cohort. Several alternative or complementary mechanisms may explain the observed benefits of our technique. The anchoring suture technique may be a key advantage beyond simple isolation effects. By establishing anchoring points along the serosal edge of the pancreatic cut surface, this technique can significantly reduce tension and shearing forces during anastomotic healing, which has been identified as a critical factor in preventing gastrointestinal anastomotic dehiscence (39). Additionally, our continuous mattress suturing technique may provide more uniform tension distribution compared to traditional interrupted sutures. Regarding the role of omental padding itself, recent studies suggest that omental tissue may promote healing through multiple mechanisms beyond simple mechanical isolation. A research conducted by Uchibori T at al. demonstrated that placement of a pedicled omental flap significantly increases vascularization of surrounding tissues while simultaneously reducing inflammatory responses. Their immunohistological and RT-PCR assessments revealed that omental tissue was associated with an increased M2/M1 macrophage phenotype ratio, decreased inflammatory marker mRNA levels, and elevated angiogenic and anti-inflammatory factor expression (40). Deng S et al. demonstrated Wrapping and isolating the modified pancreaticojejunostomy with free greater omentum can significantly reduce the incidence of POPF and related complications (41). These findings suggest that the omentum may enhance anastomotic healing by promoting vascularization and modulating inflammatory responses. Futuremore, the omental padding technique can significantly reduce the incidence of pancreatic fistula and its associated complications such as intra-abdominal infection and delayed hemorrhage (42). The innovation of this omental padding technique lies in its dual function: first, the packing effect allows pancreatic fluid to float on the omentum facilitating external drainage while eliminating cavities left after surgical dissection, thus preventing pancreatic fluid accumulation; second, the isolation effect prevents direct contact between exposed vessels and pancreatic juice, thereby protecting vessels and significantly reducing the risk of delayed postoperative hemorrhage. This omental padding technique, combined with continuous suturing and anchoring sutures, provides more comprehensive protection for subsequent pancreaticojejunal reconstruction.

Multivariate analysis identified several independent risk factors for CR-POPF: BMI ≥25 kg/m² (OR: 2.15, 95% CI: 1.03–4.52, *p* = 0.041), soft pancreatic texture (OR: 3.12, 95% CI: 1.41–6.95, *p* = 0.005), and pancreatic duct diameter < 3 mm (OR: 2.26, 95% CI: 1.06–4.83, *p* = 0.035). These factors have been widely recognized in previous studies. The intraoperative risk score proposed by Callery et al. listed soft pancreas and small pancreatic duct as primary risk factors (43). Nahm et al.'s prospective cohort study (*n* = 387) reported that soft pancreas increased CR-POPF risk by 2.9-fold, while pancreatic duct diameter < 3 mm increased risk by 2.3-fold (44). McMillan et al.'s multicenter study (*n* = 2,706) confirmed BMI ≥ 25 kg/m² as an independent risk factor (OR 1.78) (45). Notably, this study demonstrates that the modified technique independently reduces CR-POPF risk (OR 0.34) even after controlling for these known risk factors. This finding is particularly important as it suggests that the technique may provide greater benefit for high-risk patients.

In addition to reducing CR-POPF incidence, our modified technique provides other clinical benefits. Operating time was significantly shortened 287.5 ± 45.3 vs. 304.2 ± 53.6 min, *p* = 0.023), not only improving surgical efficiency but potentially reducing anesthesia-related risks. Decreased intraoperative blood loss (325 vs. 375 ml, *p* = 0.041) may reduce transfusion requirements and associated complication risks. These improvements in surgical parameters collectively promote postoperative recovery, as evidenced by faster gastrointestinal function recovery (3.2 ± 1.1 vs. 4.1 ± 1.4 days, *p* < 0.001) and earlier initiation of oral intake (4.5 ± 1.3 vs. 5.8 ± 1.8 days, *p* < 0.001). Ultimately, patients in the modified group experienced significantly shorter hospital stays (12 vs. 14 days, *p* = 0.009), which not only facilitates earlier return to normal life but may also reduce healthcare costs and nosocomial infection risks. These comprehensive improvements in perioperative indicators confirm the overall superiority of the modified technique, beyond simply reducing CR-POPF. It should be noted that after applying Bonferroni correction for multiple testing, only PJ anastomosis time, time to gastrointestinal function recovery, and time to start oral intake remained statistically significant (*p* < 0.005), suggesting other secondary outcomes should be interpreted with appropriate caution.

DGE is another common complication after PD, and we found that the modified technique also reduced its incidence (7.4% vs. 14.8%, *p* = 0.039). This may be related to the modified technique's reduction of local inflammatory response, as literature indicates an association between POPF and DGE (46–48). Our modified technique may indirectly reduce the risk of other complications by decreasing pancreatic fistula-related abdominal fluid collection and infection, which explains why the overall incidence of serious complications (Clavien-Dindo grade ≥ Ⅲ) was significantly reduced (9.6% vs. 16.4%, *p* = 0.046).

In addition to improvements in the anastomotic technique, another innovation in this study was the use of IPTW to balance baseline characteristic differences between the two groups. This methodological advantage provides a more reliable foundation for evaluating the true effect of the improved technique. Compared to traditional propensity score matching, IPTW preserves the entire sample, avoiding sample size loss due to matching, improving statistical power, and is particularly suitable for studies with limited sample sizes. Methodological research by Austin et al. confirmed that with sample sizes < 500, IPTW typically provides more robust estimates than matchin (49). Furthermore, we conducted a detailed assessment of IPTW's balancing effect, with all covariates having standardized mean differences < 0.1, indicating that weighting successfully achieved good balance.

Our results provide evidence supporting the use of improved PJ techniques in PD, especially for high-risk patients (such as those with soft pancreas, small pancreatic duct, or high BMI). The reduction in operation time and improvement in perioperative indicators suggest this technique may improve surgical efficiency and reduce medical costs. This study has several notable limitations that warrant discussion. An important limitation is the temporal separation between the c-PJ group (2020–2022) and m-BPJ group (2023–2024), which could potentially introduce confounding factors related to evolving perioperative practices. To address this concern, we performed sensitivity analyses comparing yearly outcomes (Supplementary Tables S1, S2). The results showed no significant time-dependent trends in CR-POPF rates within each technique group, with significant reduction occurring specifically at the technique transition point rather than as a gradual evolution. This pattern strongly suggests that the observed improvements were primarily attributable to the technical modification rather than to progressive optimization of perioperative management. Nevertheless, we cannot completely exclude the potential influence of unmeasured time-dependent factors in our retrospective analysis.

Another limitation relates to the potential influence of the surgeon's learning curve. Although the lead surgeon had performed over 200 pancreaticoduodenectomies prior to this study and our analysis of historical data (2018–2019) showed stable CR-POPF rates comparable to our study period c-PJ group, the accumulated experience over time could still subtly impact outcomes. However, the absence of gradual improvement within the c-PJ group over five years (2018–2022) followed by an abrupt improvement with technique change suggests technical factors rather than experience accumulation as the primary driver of improved outcomes. Furthermore, while we propose several mechanisms by which omental padding could reduce CR-POPF, we lack direct evidence (such as imaging or reoperation findings) to confirm the specific protective mechanisms. Future research incorporating systematic postoperative imaging and biomarker analysis would help elucidate these mechanisms.

Despite an effective sample size of 215 cases after IPTW, statistical power may still be limited for certain rare complications or specific subgroup analyses. Large-sample, multi-center prospective randomized controlled trials are needed to further validate the effectiveness of this improved technique, with special attention to high-risk patient subgroups (such as soft pancreas, small pancreatic duct, high BMI) to evaluate the differential effects of the improved technique in these patients. long-term follow-up studies are necessary to evaluate the impact of this technique on quality of life, long-term pancreatic function, tumor recurrence, and survival rates. Combining intraoperative objective measurements of pancreatic hardness (such as elastography or pressure measurements) with individualized risk prediction models would help select the optimal anastomotic method for patients with different risk stratifications, achieving precision treatment.

## Conclusions

This study confirms through IPTW analysis that the improved technique in PD can significantly reduce the incidence of CR-POPF, shorten operation time, promote postoperative recovery, and reduce postoperative complications. This technique has been proven to be an independent protective factor against CR-POPF, remaining effective even after controlling for known high-risk factors, suggesting it may become a preferable anastomotic method PD, especially for high-risk patients. These findings provide valuable reference for clinical practice, with the potential to improve surgical outcomes of PD, reduce patient suffering, improve quality of life, and possibly lower medical costs. Future multi-center randomized controlled trials and long-term follow-up will further validate these findings and explore the long-term benefits of the improved technique.

## Data Availability

The original contributions presented in the study are included in the article/Supplementary Material, further inquiries can be directed to the corresponding author/s.
