# Supplementary material for: Novel modified blumgart anastomosis reduces clinically relevant pancreatic fistula after pancreaticoduodenectomy: a retrospective study using inverse probability of treatment weighting
Source: Front Surg. 2025 Jun 19;12:1610561. doi: 10.3389/fsurg.2025.1610561 (PMC12222157; doi:10.3389/fsurg.2025.1610561)
Supplement: Supplementary file 1 [file Table1.docx]

Table S1. Comparison of CR-POPF Incidence Rates by Year

| Year | Technique | Number of Cases | CR-POPF, n (%) | p value* |
| --- | --- | --- | --- | --- |
| 2018-2019 | c-PJ | 59 | 9 (15.3%) | 0.645^†^ |
| 2020 | c-PJ | 43 | 5 (11.6%) | 0.758^‡^ |
| 2021 | c-PJ | 45 | 6 (13.3%) | 0.825^‡^ |
| 2022 | c-PJ | 42 | 6 (14.3%) | 0.025^§^ |
| 2023 | m-BPJ | 45 | 5 (11.1%) | 0.432^‡^ |
| 2024 | m-BPJ | 40 | 3 (7.5%) | - |
| Total | c-PJ | 130 | 17 (13.1%) | 0.408 |
| Total | m-BPJ | 85 | 8 (9.4%) |  |
| c-PJ: conventional pancreaticojejunostomy, m-BPJ: modified Blumgart pancreaticojejunostomy, CR-POPF: clinically relevant postoperative pancreatic fistula. ^*^Using Chi-square test or Fisher's exact test. ^†^Comparison with the 2020-2022 c-PJ group. ^‡^Comparison with the next year. ^§^Comparison between 2022 and 2023 (before and after technique transition). Bold indicates statistical significance (p < 0.05) | | | | |
