# Supplementary material for: Novel modified blumgart anastomosis reduces clinically relevant pancreatic fistula after pancreaticoduodenectomy: a retrospective study using inverse probability of treatment weighting
Source: Front Surg. 2025 Jun 19;12:1610561. doi: 10.3389/fsurg.2025.1610561 (PMC12222157; doi:10.3389/fsurg.2025.1610561)
Supplement: Supplementary file 2 [file Table2.docx]

Table S2. Comparison of CR-POPF Incidence Rates Before and After Technique Transition

| Time Point | Number of Cases | CR-POPF, n (%) | Blood Loss (ml), median (IQR) | PJ-time (min), mean ± SD | p value |
| --- | --- | --- | --- | --- | --- |
| 2022 (c-PJ) | 42 | 6 (14.3%) | 375 (255-515) | 28.9 ± 7.2 | 0.025 |
| 2023 (m-BPJ) | 45 | 5 (11.1%) | 315 (210-445) | 21.2 ± 5.6 |  |
| CR-POPF: clinically relevant postoperative pancreatic fistula, c-PJ: conventional pancreaticojejunostomy, m-BPJ: modified Blumgart pancreaticojejunostomy, IQR: interquartile range, SD: standard deviation, PJ: pancreaticojejunostomy. Bold indicates statistical significance (p < 0.05) | | | | | |
